# First report of *Cheiloneurus exitiosus* (Perkins, 1906) and *Helegonatopus dimorphus* (Hoffer, 1954) (Hymenoptera: Encyrtidae) from Japan, with remarks on their abundance in rice paddies

**DOI:** 10.3897/BDJ.4.e9230

**Published:** 2016-08-29

**Authors:** Toshiharu Mita, Hironobu Handa, Yoshimitsu Higashiura, George Japoshvili

**Affiliations:** ‡ Entomological Laboratory, Faculty of Agriculture, Kyushu University, Hakozaki, Higashi-ku, Fukuoka, 812-8581, Japan; § Saitama Museum of Natural History, Nagatoro, Nagatoro-machi, Saitama, 369-1305, Japan; | Citrus Promotion Center, Agricultural Technology Department, Yamaguchi Prefectural Agriculture & Forestry General Technology Center, Higashi-Agenosho, Suo-Oshima cho, Yamaguchi, 742-2805, Japan; ¶ Institute of Entomology, Agicultural University of Georgia, David Agmashenebeli Alley, 0159, Georgia

**Keywords:** Distribution, *Haplogonatopus oratorius*, host record, *Laodelphax striatellus*, parasitoid wasp

## Abstract

**Background:**

Encyrtid secondary parasitoids of Delphacidae have not been recorded in Japan. However, they may play an important role in the rice ecosystem because they can reduce the number of Dryinidae, the natural enemies of rice planthoppers.

**New information:**

We found two encyrtid species, *Cheiloneurus exitiosus* (Perkins, 1906) and *Helegonatopus dimorphus* (Hoffer, 1954), from rice paddies and the surrounding environment. *Haplogonatopus oratorius* (Westwood, 1833) and *Anteon* sp. were newly recognized as hosts of *He. dimorphus*. Parasitism of *C. exitiosus* was rare, but *He. dimorphus* was common in Kumamoto Prefecture. The sex ratio (male proportion) and clutch size of *He. dimorphus* was estimated as 0.19 and 4.95, respectively.

## Introduction

Some encyrtid wasps are known as parasitoids of Dryinidae (Guerrieri and Viggiani 2005, Olmi and Xu 2015 and Xu et al. 2013). There are two genera of encyrtids reported as parasitoids of Dryinidae, namely, *Cheiloneurus* Westwood and *Helegonatopus* Perkins. Hitherto, *Helegonatopus* is considered the specialist of Dryinidae. Among these, *H. dimorphus* (Hoffer, 1954) has been widely recorded from Europe to Sakhalin (Noyes 2016). This species exhibits a peculiar sexual dimorphism. The head of the male is compressed laterally, whereas that of the female is unmodified. As for *Cheiloneurus*, *C. exitiosus* (Perkins, 1906) is recorded widely from South Asia to Oceania (Xu et al. 2013). As is often the case with small parasitoid wasps, a substantial number of Encyrtidae show a cosmopolitan distribution. Furthermore, some of their primary hosts such as rice planthoppers are known as long-distance migrants (Otuka 2013), and dryinid larvae can also be transported (Mita et al. 2013). It is possible that those primary hosts carry not only dryinid larvae, but also their hyperparasitoids. In East Asian countries, the secondary parasitoid of rice planthoppers has seldom been investigated. However, a consideration of their distribution range and the host's dispersal ability suggests they should be found widely in East Asian countries including Japan, and they may play an indispensable role in the rice ecosystem. We herein report the occurrence of these encyrtid wasps in Japan, and present results of a preliminary survey of their field abundance.

## Materials and methods

Materials used in the study are preserved in 99.5% ethanol. They were mounted on pieces of cardboard or glass slides when necessary. Original pictures were taken by a digital camera (Olympus E-5) attached to an Olympus SZX10 stereomicroscope. Photo images were processed using image-stacking software (Combine ZP). Materials are deposited in the Entomological Laboratory, Faculty of Agriculture, Kyushu University (ELKU). Distribution and host records were obtained from Guerrieri and Viggiani (2005), Xu et al. (2013) and Olmi and Xu (2015).

Some encyrtid wasps collected before 2014 could not be separated into each host individual. To confirm the number of encyrtid wasps per dryinid larva, the parasitism ratio, and the sex ratio of encyrtid hyperparasitoids, specimens of *Laodelphax striatellus* (Fallén, 1826) parasitized by Dryinidae and dryinid cocoons on leaves were collected from rice paddies in Kumamoto and Kagoshima prefectures on Kyushu Island during September 17–28, 2014 (Table 1). Two other species of rice planthoppers, *Nilaparvata lugens* (Stål, 1854) and *Sogatella furcifera* (Horváth, 1899), were seldom collected during the survey. Cocoons were additionally collected at Suya from September to October 2014. Parasitized planthoppers were reared separately in small glass tubes plugged by cotton with a stem of rice. Field cocoons were similarly treated, but a stem was not inserted. They were kept in an incubator (25°C, 16L/8D) or the laboratory at room temperature. When no parasitoid emerged, the cocoon was dissected and any dead wasps were identified.

## Taxon treatments

### Cheiloneurus exitiosus

(Perkins, 1906)

#### Materials

- **Type status:** Other material. **Occurrence:** recordedBy: Toshiharu Mita; individualCount: 5; sex: 1 male, 4 females; lifeStage: adult; **Location:** country: Japan; stateProvince: Kumamoto; locality: rice paddy, Hida, Kita-ku, Kumamoto, Japan; decimalLatitude: 32.849; decimalLongitude: 130.720; **Identification:** identifiedBy: Toshiharu Mita; dateIdentified: 2014; **Event:** samplingProtocol: collecting coccoon of Haplogonatopus oratorius; eventDate: 2014-09-20/2014-10; **Record Level:** modified: 2015-12-05; institutionID: ELKU
- **Type status:** Other material. **Occurrence:** recordedBy: Toshiharu Mita; individualCount: 5; sex: 1 male 4 females; lifeStage: adult; **Location:** country: Japan; stateProvince: Nagasaki; locality: rice paddy, Nagasaki Plant Protection Office, Kobunakoshi-machi, Nagasaki, Japan; decimalLatitude: 32.837; decimalLongitude: 130.024; **Identification:** identifiedBy: Toshiharu Mita; dateIdentified: 2014; **Event:** samplingProtocol: sweeping of parasitised Sogatella furcifera by Haplogonatopus apicalis and rearing of adult wasps; eventDate: 2009-08-26/2009-09; **Record Level:** modified: 2015-12-05; institutionID: ELKU
- **Type status:** Other material. **Occurrence:** recordedBy: Toshiharu Mita; individualCount: 1; sex: 1 male; lifeStage: adult; **Location:** country: Japan; stateProvince: Nagasaki; locality: rice paddy, Nagasaki Plant Protection Office, Kobunakoshi-machi, Nagasaki, Japan; decimalLatitude: 32.837; decimalLongitude: 130.024; **Identification:** identifiedBy: Toshiharu Mita; dateIdentified: 2014; **Event:** samplingProtocol: sweeping of parasitised Sogatella furcifera by Haplogonatopus apicalis and rearing of adult wasp; eventDate: 2009-08-26/2009-09; **Record Level:** modified: 2015-12-25; institutionID: ELKU

#### Distribution

American Samoa, Australia, China, Fiji, Guam, India, Malaysia, Philippines, Western Samoa (Noyes 2016), Japan, **new record**: Kyushu (Figs 1, 2​).

##### Host

Palaearctic Region: *Gonatopus camelinus* Kieffer, 1904 (Spain). Oriental Region: *Haplogonatopus apicalis* Perkins, 1905 (Malaya; Japan); *H. oratorius* (Westwood, 1833) (China; Japan); *G. flavifemur* (Esaki & Hashimoto, 1932) (Philippines); *G. nigricans* (Perkins, 1905) (Malaya); *G. nudus* (Perkins, 1912) (India).

### Helegoantopus dimorphus

(Hoffer, 1954)

#### Materials

- **Type status:** Other material. **Occurrence:** recordedBy: Hironobu Handa; individualCount: 5; sex: 1 male, 4 females; lifeStage: adult; **Location:** country: Japan; stateProvince: Kumamoto; locality: rice paddy, Shisui-machi, Kikuchi-shi, Kumamoto, Japan; decimalLatitude: 32.927; decimalLongitude: 130.766; **Identification:** identifiedBy: Hironobu Handa; dateIdentified: 2014; **Event:** samplingProtocol: collecting coccoon of Haplogonatopus oratorius and rearing of adult wasp; eventDate: 2014-09-21/2014-10-05; **Record Level:** modified: 2015-12-05; institutionID: ELKU
- **Type status:** Other material. **Occurrence:** recordedBy: Hironobu Handa; individualCount: 5; sex: 1 male, 4 females; lifeStage: adult; **Location:** country: Japan; stateProvince: Kumamoto; locality: rice paddy, Shisui-machi, Kikuchi-shi, Kumamoto, Japan; decimalLatitude: 32.927; decimalLongitude: 130.766; **Identification:** identifiedBy: Hironobu Handa; dateIdentified: 2014; **Event:** samplingProtocol: collecting coccoon of Haplogonatopus oratorius and rearing of adult wasp; eventDate: 2014-09-21/2014-10-09; **Record Level:** modified: 2015-12-05; institutionID: ELKU
- **Type status:** Other material. **Occurrence:** recordedBy: Hironobu Handa; individualCount: 3; sex: 1 male, 2 females; lifeStage: adult; **Location:** country: Japan; stateProvince: Kumamoto; locality: rice paddy, Hida, Kita-ku, Kumamoto, Japan; decimalLatitude: 32.849; decimalLongitude: 130.720; **Identification:** identifiedBy: Hironobu Handa; dateIdentified: 2014; **Event:** samplingProtocol: sweeping of parsitised nymph host planthopper (Laodelphax striatellus) and rearing of adult wasp; eventDate: 2014-09-18/2014-10-05; **Record Level:** modified: 2015-12-25; institutionID: ELKU
- **Type status:** Other material. **Occurrence:** recordedBy: Hironobu Handa; individualCount: 3; sex: 1 male, 2 females; lifeStage: adult; **Location:** country: Japan; stateProvince: Kumamoto; locality: rice paddy, Hida, Kita-ku, Kumamoto, Japan; decimalLatitude: 32.849; decimalLongitude: 130.720; **Identification:** identifiedBy: Hironobu Handa; dateIdentified: 2014; **Event:** samplingProtocol: sweeping of parsitised brachipterous adult host planthopper (Laodelphax striatellus) and rearing of adult wasp; eventDate: 2014-09-18/2014-10-04; **Record Level:** modified: 2015-12-25; institutionID: ELKU
- **Type status:** Other material. **Occurrence:** recordedBy: Hironobu Handa; individualCount: 5; sex: 1 male, 4 females; lifeStage: adult; **Location:** country: Japan; stateProvince: Kumamoto; locality: rice paddy, Hida, Kita-ku, Kumamoto, Japan; decimalLatitude: 32.849; decimalLongitude: 130.720; **Identification:** identifiedBy: Hironobu Handa; dateIdentified: 2014; **Event:** samplingProtocol: collecting coccoon of Haplogonatopus oratorius and rearing of adult wasp; eventDate: 2014-09-18/2014-09-27; **Record Level:** modified: 2015-12-25; institutionID: ELKU
- **Type status:** Other material. **Occurrence:** recordedBy: Hironobu Handa; individualCount: 5; sex: 1 male, 4 females; lifeStage: adult; **Location:** country: Japan; stateProvince: Kumamoto; locality: rice paddy, Hida, Kita-ku, Kumamoto, Japan; decimalLatitude: 32.849; decimalLongitude: 130.720; **Identification:** identifiedBy: Hironobu Handa; dateIdentified: 2014; **Event:** samplingProtocol: collecting coccoon of Haplogonatopus oratorius and rearing of adult wasp; eventDate: 2014-09-18/2014-10-04; **Record Level:** modified: 2015-12-25; institutionID: ELKU
- **Type status:** Other material. **Occurrence:** recordedBy: Hironobu Handa; individualCount: 4; sex: 1 male, 3 females; lifeStage: adult; **Location:** country: Japan; stateProvince: Kumamoto; locality: rice paddy, Hida, Kita-ku, Kumamoto, Japan; decimalLatitude: 32.849; decimalLongitude: 130.720; **Identification:** identifiedBy: Hironobu Handa; dateIdentified: 2014; **Event:** samplingProtocol: collecting coccoon of Haplogonatopus oratorius and rearing of adult wasp; eventDate: 2014-09-20/2014-09-30; **Record Level:** modified: 2015-12-25; institutionID: ELKU
- **Type status:** Other material. **Occurrence:** recordedBy: Hironobu Handa; individualCount: 3; sex: 3 females; lifeStage: adult; **Location:** country: Japan; stateProvince: Kumamoto; locality: rice paddy, Hida, Kita-ku, Kumamoto, Japan; decimalLatitude: 32.849; decimalLongitude: 130.720; **Identification:** identifiedBy: Hironobu Handa; dateIdentified: 2014; **Event:** samplingProtocol: collecting coccoon of Haplogonatopus oratorius and rearing of adult wasp; eventDate: 2014-09-20/2014-09-27; **Record Level:** modified: 2015-12-25; institutionID: ELKU
- **Type status:** Other material. **Occurrence:** recordedBy: Hironobu Handa; individualCount: 5; sex: 1 male, 4 females; lifeStage: adult; **Location:** country: Japan; stateProvince: Kumamoto; locality: rice paddy, Hida, Kita-ku, Kumamoto, Japan; decimalLatitude: 32.849; decimalLongitude: 130.720; **Identification:** identifiedBy: Hironobu Handa; dateIdentified: 2014; **Event:** samplingProtocol: collecting coccoon of Haplogonatopus oratorius and rearing of adult wasp; eventDate: 2014-09-20/2014-10-05; **Record Level:** modified: 2015-12-25; institutionID: ELKU
- **Type status:** Other material. **Occurrence:** recordedBy: Toshiharu Mita; individualCount: 6; sex: 1 male, 5 females; lifeStage: adult; **Location:** country: Japan; stateProvince: Kumamoto; locality: rice paddy, NARO Kyushu Okinawa Agricultural Research Center, Koshi, Kumamoto, Japan; decimalLatitude: 32.876; decimalLongitude: 130.738; **Identification:** identifiedBy: Toshiharu Mita; dateIdentified: 2014; **Event:** samplingProtocol: adult wasp emerged from coccoon of Haplogonatopus oratorius; eventDate: 2014-08-30/2014-09-04; **Record Level:** modified: 2015-12-25; institutionID: ELKU
- **Type status:** Other material. **Occurrence:** recordedBy: Toshiharu Mita; individualCount: 6; sex: 6 females; lifeStage: adult; **Location:** country: Japan; stateProvince: Kumamoto; locality: rice paddy, NARO Kyushu Okinawa Agricultural Research Center, Koshi, Kumamoto, Japan; decimalLatitude: 32.876; decimalLongitude: 130.738; **Identification:** identifiedBy: Toshiharu Mita; dateIdentified: 2014; **Event:** samplingProtocol: adult wasp emerged from coccoon of Haplogonatopus oratorius; eventDate: 2014-08-30/2014-09-04; **Record Level:** modified: 2015-12-25; institutionID: ELKU
- **Type status:** Other material. **Occurrence:** recordedBy: Toshiharu Mita; individualCount: 4; sex: 1 male, 3 females; lifeStage: adult; **Location:** country: Japan; stateProvince: Kumamoto; locality: rice paddy, NARO Kyushu Okinawa Agricultural Research Center, Koshi, Kumamoto, Japan; decimalLatitude: 32.876; decimalLongitude: 130.738; **Identification:** identifiedBy: Toshiharu Mita; dateIdentified: 2014; **Event:** samplingProtocol: adult wasp emerged from coccoon of Haplogonatopus oratorius; eventDate: 2014-08-30/2014-09-06; **Record Level:** modified: 2015-12-25; institutionID: ELKU
- **Type status:** Other material. **Occurrence:** recordedBy: Toshiharu Mita; individualCount: 4; sex: 1 male, 3 females; lifeStage: adult; **Location:** country: Japan; stateProvince: Kumamoto; locality: rice paddy, NARO Kyushu Okinawa Agricultural Research Center, Koshi, Kumamoto, Japan; decimalLatitude: 32.876; decimalLongitude: 130.738; **Identification:** identifiedBy: Toshiharu Mita; dateIdentified: 2014; **Event:** samplingProtocol: adult wasp emerged from coccoon of Haplogonatopus oratorius; eventDate: 2014-08-30/2014-09-07; **Record Level:** modified: 2015-12-25; institutionID: ELKU
- **Type status:** Other material. **Occurrence:** recordedBy: Toshiharu Mita; individualCount: 3; sex: 1 male, 2 females; lifeStage: adult; **Location:** country: Japan; stateProvince: Kumamoto; locality: rice paddy, NARO Kyushu Okinawa Agricultural Research Center, Koshi, Kumamoto, Japan; decimalLatitude: 32.876; decimalLongitude: 130.738; **Identification:** identifiedBy: Toshiharu Mita; dateIdentified: 2014; **Event:** samplingProtocol: adult wasp emerged from coccoon of Haplogonatopus oratorius; eventDate: 2014-08-30/2014-09-08; **Record Level:** modified: 2015-12-25; institutionID: ELKU
- **Type status:** Other material. **Occurrence:** recordedBy: Toshiharu Mita; individualCount: 5; sex: 1 male, 4 females; lifeStage: adult; **Location:** country: Japan; stateProvince: Kumamoto; locality: rice paddy, NARO Kyushu Okinawa Agricultural Research Center, Koshi, Kumamoto, Japan; decimalLatitude: 32.876; decimalLongitude: 130.738; **Identification:** identifiedBy: Toshiharu Mita; dateIdentified: 2014; **Event:** samplingProtocol: adult wasp emerged from coccoon of Haplogonatopus oratorius; eventDate: 2014-08-31/2014-09-11; **Record Level:** modified: 2015-12-25; institutionID: ELKU
- **Type status:** Other material. **Occurrence:** recordedBy: Toshiharu Mita; individualCount: 4; sex: 1 male, 3 females; lifeStage: adult; **Location:** country: Japan; stateProvince: Kumamoto; locality: rice paddy, NARO Kyushu Okinawa Agricultural Research Center, Koshi, Kumamoto, Japan; decimalLatitude: 32.876; decimalLongitude: 130.738; **Identification:** identifiedBy: Toshiharu Mita; dateIdentified: 2014; **Event:** samplingProtocol: adult wasp emerged from coccoon of Haplogonatopus oratorius; eventDate: 2014-08-31/2014-09-11; **Record Level:** modified: 2015-12-25; institutionID: ELKU
- **Type status:** Other material. **Occurrence:** recordedBy: Toshiharu Mita; individualCount: 8; sex: 2 males, 6 females; lifeStage: adult; **Location:** country: Japan; stateProvince: Kumamoto; locality: rice paddy, NARO Kyushu Okinawa Agricultural Research Center, Koshi, Kumamoto, Japan; decimalLatitude: 32.876; decimalLongitude: 130.738; **Identification:** identifiedBy: Toshiharu Mita; dateIdentified: 2014; **Event:** samplingProtocol: adult wasp emerged from coccoon of Haplogonatopus oratorius; eventDate: 2014-08-31/2014-09-11; **Record Level:** modified: 2015-12-25; institutionID: ELKU
- **Type status:** Other material. **Occurrence:** recordedBy: Toshiharu Mita; individualCount: 6; sex: 1 male, 5 females; lifeStage: adult; **Location:** country: Japan; stateProvince: Kumamoto; locality: rice paddy, NARO Kyushu Okinawa Agricultural Research Center, Koshi, Kumamoto, Japan; decimalLatitude: 32.876; decimalLongitude: 130.738; **Identification:** identifiedBy: Toshiharu Mita; dateIdentified: 2014; **Event:** samplingProtocol: adult wasp emerged from coccoon of Haplogonatopus oratorius; eventDate: 2014-08-31/2014-09-11; **Record Level:** modified: 2015-12-25; institutionID: ELKU
- **Type status:** Other material. **Occurrence:** recordedBy: Toshiharu Mita; individualCount: 5; sex: 1 male, 4 females; lifeStage: adult; **Location:** country: Japan; stateProvince: Kumamoto; locality: rice paddy, NARO Kyushu Okinawa Agricultural Research Center, Koshi, Kumamoto, Japan; decimalLatitude: 32.876; decimalLongitude: 130.738; **Identification:** identifiedBy: Toshiharu Mita; dateIdentified: 2014; **Event:** samplingProtocol: adult wasp emerged from coccoon of Haplogonatopus oratorius; eventDate: 2014-08-31/2014-09-11; **Record Level:** modified: 2015-12-25; institutionID: ELKU
- **Type status:** Other material. **Occurrence:** recordedBy: Toshiharu Mita; individualCount: 6; sex: 1 male, 5 females; lifeStage: adult; **Location:** country: Japan; stateProvince: Kumamoto; locality: rice paddy, NARO Kyushu Okinawa Agricultural Research Center, Koshi, Kumamoto, Japan; decimalLatitude: 32.876; decimalLongitude: 130.738; **Identification:** identifiedBy: Toshiharu Mita; dateIdentified: 2014; **Event:** samplingProtocol: adult wasp emerged from coccoon of Haplogonatopus oratorius; eventDate: 2014-09-01/2014-09-12; **Record Level:** modified: 2015-12-25; institutionID: ELKU
- **Type status:** Other material. **Occurrence:** recordedBy: Toshiharu Mita; individualCount: 9; sex: 3 males, 6 females; lifeStage: adult; **Location:** country: Japan; stateProvince: Kumamoto; locality: rice paddy, NARO Kyushu Okinawa Agricultural Research Center, Koshi, Kumamoto, Japan; decimalLatitude: 32.876; decimalLongitude: 130.738; **Identification:** identifiedBy: Toshiharu Mita; dateIdentified: 2014; **Event:** samplingProtocol: adult wasp emerged from coccoon of Haplogonatopus oratorius; eventDate: 2014-09-01/2014-09-12; **Record Level:** modified: 2015-12-25; institutionID: ELKU
- **Type status:** Other material. **Occurrence:** recordedBy: Toshiharu Mita; individualCount: 7; sex: 3 males, 4 females; lifeStage: adult; **Location:** country: Japan; stateProvince: Kumamoto; locality: rice paddy, NARO Kyushu Okinawa Agricultural Research Center, Koshi, Kumamoto, Japan; decimalLatitude: 32.876; decimalLongitude: 130.738; **Identification:** identifiedBy: Toshiharu Mita; dateIdentified: 2014; **Event:** samplingProtocol: adult wasp emerged from coccoon of Haplogonatopus oratorius; eventDate: 2014-09-01/2014-09-12; **Record Level:** modified: 2015-12-25; institutionID: ELKU
- **Type status:** Other material. **Occurrence:** recordedBy: Toshiharu Mita; individualCount: 4; sex: 1 male, 3 females; lifeStage: adult; **Location:** country: Japan; stateProvince: Kumamoto; locality: rice paddy, NARO Kyushu Okinawa Agricultural Research Center, Koshi, Kumamoto, Japan; decimalLatitude: 32.876; decimalLongitude: 130.738; **Identification:** identifiedBy: Toshiharu Mita; dateIdentified: 2014; **Event:** samplingProtocol: adult wasp emerged from coccoon of Haplogonatopus oratorius; eventDate: 2014-09-01/2014-09-12; **Record Level:** modified: 2015-12-25; institutionID: ELKU
- **Type status:** Other material. **Occurrence:** recordedBy: Toshiharu Mita; individualCount: 5; sex: 1 male, 4 females; lifeStage: adult; **Location:** country: Japan; stateProvince: Kumamoto; locality: rice paddy, NARO Kyushu Okinawa Agricultural Research Center, Koshi, Kumamoto, Japan; decimalLatitude: 32.876; decimalLongitude: 130.738; **Identification:** identifiedBy: Toshiharu Mita; dateIdentified: 2014; **Event:** samplingProtocol: adult wasp emerged from coccoon of Haplogonatopus oratorius; eventDate: 2014-09/2014-09-17; **Record Level:** modified: 2015-12-25; institutionID: ELKU
- **Type status:** Other material. **Occurrence:** recordedBy: Toshiharu Mita; individualCount: 6; sex: 1 male, 5 females; lifeStage: adult; **Location:** country: Japan; stateProvince: Kumamoto; locality: rice paddy, NARO Kyushu Okinawa Agricultural Research Center, Koshi, Kumamoto, Japan; decimalLatitude: 32.876; decimalLongitude: 130.738; **Identification:** identifiedBy: Toshiharu Mita; dateIdentified: 2014; **Event:** samplingProtocol: adult wasp emerged from coccoon of Haplogonatopus oratorius; eventDate: 2014-09/2014-09-17; **Record Level:** modified: 2015-12-25; institutionID: ELKU
- **Type status:** Other material. **Occurrence:** recordedBy: Toshiharu Mita; individualCount: 4; sex: 1 male, 3 females; lifeStage: adult; **Location:** country: Japan; stateProvince: Kumamoto; locality: rice paddy, NARO Kyushu Okinawa Agricultural Research Center, Koshi, Kumamoto, Japan; decimalLatitude: 32.876; decimalLongitude: 130.738; **Identification:** identifiedBy: Toshiharu Mita; dateIdentified: 2014; **Event:** samplingProtocol: adult wasp emerged from coccoon of Haplogonatopus oratorius; eventDate: 2014-09/2014-09-17; **Record Level:** modified: 2015-12-25; institutionID: ELKU
- **Type status:** Other material. **Occurrence:** recordedBy: Toshiharu Mita; individualCount: 5; sex: 1 male, 4 females; lifeStage: adult; **Location:** country: Japan; stateProvince: Kumamoto; locality: rice paddy, NARO Kyushu Okinawa Agricultural Research Center, Koshi, Kumamoto, Japan; decimalLatitude: 32.876; decimalLongitude: 130.738; **Identification:** identifiedBy: Toshiharu Mita; dateIdentified: 2014; **Event:** samplingProtocol: adult wasp emerged from coccoon of Haplogonatopus oratorius; eventDate: 2014-09/2014-09-20; **Record Level:** modified: 2015-12-25; institutionID: ELKU
- **Type status:** Other material. **Occurrence:** recordedBy: Toshiharu Mita; individualCount: 6; sex: 1 male, 5 females; lifeStage: adult; **Location:** country: Japan; stateProvince: Kumamoto; locality: rice paddy, NARO Kyushu Okinawa Agricultural Research Center, Koshi, Kumamoto, Japan; decimalLatitude: 32.876; decimalLongitude: 130.738; **Identification:** identifiedBy: Toshiharu Mita; dateIdentified: 2014; **Event:** samplingProtocol: adult wasp emerged from coccoon of Haplogonatopus oratorius; eventDate: 2014-09/2014-09-20; **Record Level:** modified: 2015-12-25; institutionID: ELKU
- **Type status:** Other material. **Occurrence:** recordedBy: Toshiharu Mita; individualCount: 5; sex: 1 male, 4 females; lifeStage: adult; **Location:** country: Japan; stateProvince: Kumamoto; locality: rice paddy, NARO Kyushu Okinawa Agricultural Research Center, Koshi, Kumamoto, Japan; decimalLatitude: 32.876; decimalLongitude: 130.738; **Identification:** identifiedBy: Toshiharu Mita; dateIdentified: 2014; **Event:** samplingProtocol: adult wasp emerged from coccoon of Haplogonatopus oratorius; eventDate: 2014-09/2014-09-26; **Record Level:** modified: 2015-12-25; institutionID: ELKU
- **Type status:** Other material. **Occurrence:** recordedBy: Toshiharu Mita; individualCount: 6; sex: 1 male, 5 females; lifeStage: adult; **Location:** country: Japan; stateProvince: Kumamoto; locality: rice paddy, NARO Kyushu Okinawa Agricultural Research Center, Koshi, Kumamoto, Japan; decimalLatitude: 32.876; decimalLongitude: 130.738; **Identification:** identifiedBy: Toshiharu Mita; dateIdentified: 2014; **Event:** samplingProtocol: adult wasp emerged from coccoon of Haplogonatopus oratorius; eventDate: 2014-09/2014-10-01; **Record Level:** modified: 2015-12-25; institutionID: ELKU
- **Type status:** Other material. **Occurrence:** recordedBy: Toshiharu Mita; individualCount: 5; sex: 5 females; lifeStage: adult; **Location:** country: Japan; stateProvince: Kumamoto; locality: rice paddy, NARO Kyushu Okinawa Agricultural Research Center, Koshi, Kumamoto, Japan; decimalLatitude: 32.876; decimalLongitude: 130.738; **Identification:** identifiedBy: Toshiharu Mita; dateIdentified: 2014; **Event:** samplingProtocol: adult wasp emerged from coccoon of Haplogonatopus oratorius; eventDate: 2014-09/2014-09-10; **Record Level:** modified: 2015-12-25; institutionID: ELKU
- **Type status:** Other material. **Occurrence:** recordedBy: Toshiharu Mita; individualCount: 5; sex: 5 females; lifeStage: adult; **Location:** country: Japan; stateProvince: Kumamoto; locality: rice paddy, NARO Kyushu Okinawa Agricultural Research Center, Koshi, Kumamoto, Japan; decimalLatitude: 32.876; decimalLongitude: 130.738; **Identification:** identifiedBy: Toshiharu Mita; dateIdentified: 2014; **Event:** samplingProtocol: adult wasp emerged from coccoon of Haplogonatopus oratorius; eventDate: 2014-09/2014-09-10; **Record Level:** modified: 2015-12-25; institutionID: ELKU
- **Type status:** Other material. **Occurrence:** recordedBy: Toshiharu Mita; individualCount: 4; sex: 4 females; lifeStage: adult; **Location:** country: Japan; stateProvince: Kumamoto; locality: rice paddy, NARO Kyushu Okinawa Agricultural Research Center, Koshi, Kumamoto, Japan; decimalLatitude: 32.876; decimalLongitude: 130.738; **Identification:** identifiedBy: Toshiharu Mita; dateIdentified: 2014; **Event:** samplingProtocol: adult wasp emerged from coccoon of Haplogonatopus oratorius; eventDate: 2014-09/2014-09-12; **Record Level:** modified: 2015-12-25; institutionID: ELKU
- **Type status:** Other material. **Occurrence:** recordedBy: Toshiharu Mita; individualCount: 3; sex: 3 females; lifeStage: adult; **Location:** country: Japan; stateProvince: Kumamoto; locality: rice paddy, NARO Kyushu Okinawa Agricultural Research Center, Koshi, Kumamoto, Japan; decimalLatitude: 32.876; decimalLongitude: 130.738; **Identification:** identifiedBy: Toshiharu Mita; dateIdentified: 2014; **Event:** samplingProtocol: adult wasp emerged from coccoon of Haplogonatopus oratorius; eventDate: 2014-09/2014-09-12; **Record Level:** modified: 2015-12-25; institutionID: ELKU
- **Type status:** Other material. **Occurrence:** recordedBy: Toshiharu Mita; individualCount: 5; sex: 5 females; lifeStage: adult; **Location:** country: Japan; stateProvince: Kumamoto; locality: rice paddy, NARO Kyushu Okinawa Agricultural Research Center, Koshi, Kumamoto, Japan; decimalLatitude: 32.876; decimalLongitude: 130.738; **Identification:** identifiedBy: Toshiharu Mita; dateIdentified: 2014; **Event:** samplingProtocol: adult wasp emerged from coccoon of Haplogonatopus oratorius; eventDate: 2014-09/2014-09-17; **Record Level:** modified: 2015-12-25; institutionID: ELKU
- **Type status:** Other material. **Occurrence:** recordedBy: Toshiharu Mita; individualCount: 3; sex: 3 females; lifeStage: adult; **Location:** country: Japan; stateProvince: Kumamoto; locality: rice paddy, NARO Kyushu Okinawa Agricultural Research Center, Koshi, Kumamoto, Japan; decimalLatitude: 32.876; decimalLongitude: 130.738; **Identification:** identifiedBy: Toshiharu Mita; dateIdentified: 2014; **Event:** samplingProtocol: adult wasp emerged from coccoon of Haplogonatopus oratorius; eventDate: 2014-09/2014-09-17; **Record Level:** modified: 2015-12-25; institutionID: ELKU
- **Type status:** Other material. **Occurrence:** recordedBy: Toshiharu Mita; individualCount: 4; sex: 4 females; lifeStage: adult; **Location:** country: Japan; stateProvince: Kumamoto; locality: rice paddy, NARO Kyushu Okinawa Agricultural Research Center, Koshi, Kumamoto, Japan; decimalLatitude: 32.876; decimalLongitude: 130.738; **Identification:** identifiedBy: Toshiharu Mita; dateIdentified: 2014; **Event:** samplingProtocol: adult wasp emerged from coccoon of Haplogonatopus oratorius; eventDate: 2014-09/2014-09-26; **Record Level:** modified: 2015-12-25; institutionID: ELKU
- **Type status:** Other material. **Occurrence:** recordedBy: Toshiharu Mita; individualCount: 7; sex: 3 males, 4 females; lifeStage: adult; **Location:** country: Japan; stateProvince: Kumamoto; locality: rice paddy, NARO Kyushu Okinawa Agricultural Research Center, Koshi, Kumamoto, Japan; decimalLatitude: 32.876; decimalLongitude: 130.738; **Identification:** identifiedBy: Toshiharu Mita; dateIdentified: 2014; **Event:** samplingProtocol: collecting coccoon of Haplogonatopus oratorius and examined dead adult wasp; eventDate: 2014-09-20/2014-10; **Record Level:** modified: 2015-12-25; institutionID: ELKU
- **Type status:** Other material. **Occurrence:** recordedBy: Toshiharu Mita; individualCount: 1; sex: 1 male; lifeStage: adult; **Location:** country: Japan; stateProvince: Kumamoto; locality: rice paddy, NARO Kyushu Okinawa Agricultural Research Center, Koshi, Kumamoto, Japan; decimalLatitude: 32.876; decimalLongitude: 130.738; **Identification:** identifiedBy: Toshiharu Mita; dateIdentified: 2014; **Event:** samplingProtocol: collecting coccoon of Haplogonatopus oratorius and examined dead adult wasp; eventDate: 2014-10-02; **Record Level:** modified: 2015-12-25; institutionID: ELKU
- **Type status:** Other material. **Occurrence:** recordedBy: Toshiharu Mita; individualCount: 1; sex: 1 male; lifeStage: adult; **Location:** country: Japan; stateProvince: Kumamoto; locality: rice paddy, NARO Kyushu Okinawa Agricultural Research Center, Koshi, Kumamoto, Japan; decimalLatitude: 32.876; decimalLongitude: 130.738; **Identification:** identifiedBy: Toshiharu Mita; dateIdentified: 2014; **Event:** samplingProtocol: collecting coccoon of Haplogonatopus oratorius and examined dead adult wasp; eventDate: 2014-10-02; **Record Level:** modified: 2015-12-25; institutionID: ELKU
- **Type status:** Other material. **Occurrence:** recordedBy: Toshiharu Mita; individualCount: 1; sex: 1 male; lifeStage: adult; **Location:** country: Japan; stateProvince: Kumamoto; locality: rice paddy, NARO Kyushu Okinawa Agricultural Research Center, Koshi, Kumamoto, Japan; decimalLatitude: 32.876; decimalLongitude: 130.738; **Identification:** identifiedBy: Toshiharu Mita; dateIdentified: 2014; **Event:** samplingProtocol: collecting coccoon of Haplogonatopus oratorius and examined dead adult wasp; eventDate: 2014-10-02; **Record Level:** modified: 2015-12-25; institutionID: ELKU
- **Type status:** Other material. **Occurrence:** recordedBy: Toshiharu Mita; individualCount: 4; sex: 4 females; lifeStage: adult; **Location:** country: Japan; stateProvince: Kumamoto; locality: rice paddy, Kamo-cho, Yamaga-shi, Kumamoto, Japan; **Identification:** identifiedBy: Toshiharu Mita; dateIdentified: 2014; **Event:** samplingProtocol: collecting coccoon of rice planthopper and rearing of adult wasps; eventDate: 2009-08-19/2009-08; **Record Level:** modified: 2015-12-25; institutionID: ELKU
- **Type status:** Other material. **Occurrence:** recordedBy: Atsuhito Sakai; individualCount: 3; sex: 3 females; lifeStage: adult; **Location:** country: Japan; stateProvince: Kanagawa; locality: rice paddy, Funako, Atsugi-shi, Kanagawa, Japan; decimalLatitude: 35.434; decimalLongitude: 139.351; **Identification:** identifiedBy: Toshiharu Mita; dateIdentified: 2014; **Event:** samplingProtocol: sweeping of parsitised host planthopper (Laodelphax striatellus) and rearing of adult wasps; eventDate: 2010-09-17/2010-10; **Record Level:** modified: 2015-12-25; institutionID: ELKU
- **Type status:** Other material. **Occurrence:** recordedBy: Atsuhito Sakai; individualCount: 5; sex: 2 males, 3 females; lifeStage: adult; **Location:** country: Japan; stateProvince: Kanagawa; locality: rice paddy, Funako, Atsugi-shi, Kanagawa, Japan; decimalLatitude: 35.434; decimalLongitude: 139.351; **Identification:** identifiedBy: Toshiharu Mita; dateIdentified: 2014; **Event:** samplingProtocol: sweeping of parsitised host planthopper (Laodelphax striatellus) and rearing of adult wasps; eventDate: 2010-09-15/2010-10; **Record Level:** modified: 2015-12-25; institutionID: ELKU
- **Type status:** Other material. **Occurrence:** recordedBy: Toshiharu Mita; individualCount: 42; sex: 11 males, 31 females; lifeStage: adult; **Location:** country: Japan; stateProvince: Nagasaki; locality: rice paddy, Nagasaki Plant Protection Office, Kobunakoshi-machi, Nagasaki, Japan; decimalLatitude: 32.837; decimalLongitude: 130.024; **Identification:** identifiedBy: Toshiharu Mita; dateIdentified: 2014; **Event:** samplingProtocol: sweeping of parasitised Sogatella furcifera by Haplogonatopus apicalis and rearing of adult was, pmultipul individuals of hosts kept in same tube; eventDate: 2009-08-26/2009-09; **Record Level:** modified: 2015-12-25; institutionID: ELKU
- **Type status:** Other material. **Occurrence:** recordedBy: Naomichi Ohara; individualCount: 4; sex: 3 males, 1 female; lifeStage: adult; **Location:** country: Japan; stateProvince: Saitama; locality: grassland, Ooasou-kouen, Kumagaya-shi, Saitama, Japan; decimalLatitude: 36.143; decimalLongitude: 139.348; **Identification:** identifiedBy: Toshiharu Mita; dateIdentified: 2014; **Event:** samplingProtocol: sweeping of parasitised Hecalus sp. by Anteon sp. and rearing adult wasp; eventDate: 2008-10-20/2008-11; **Record Level:** modified: 2015-12-25; institutionID: ELKU
- **Type status:** Other material. **Occurrence:** recordedBy: Toshiharu Mita; individualCount: 2; sex: 2 females; lifeStage: adult; **Location:** country: Japan; stateProvince: Fukuoka; locality: fallow field, Sasaguri-machi, Fukuoka, Japan; **Identification:** identifiedBy: Toshiharu Mita; dateIdentified: 2014; **Event:** samplingProtocol: collecting of cocoon of Haplogonatopus sp. and rearing of adult wasp; eventDate: 2009-08-11/2009-08; **Record Level:** modified: 2015-12-25; institutionID: ELKU

#### Distribution

Widely distributed from western Palaearctic countries to Mongolia (Noyes 2016), Japan, **new record**: Honshu, Kyushu (Figs 3, 4).

#### Biology

##### Host

Palaearctic Region: *Anteon* sp., **new record** (Japan); *Haplogonatopus oratorius* (Westwood, 1833), **new record** (Japan); *Gonatopus clavipes* (Thunberg, 1827) (Italy); *G. solidus* (Haupt, 1938) (Italy); *G. formicicolus* (Richards, 1939) (Italy); *G. pallidus* (Ceballos, 1927) (Sweden); *G. rosellae* (Currado & Olmi, 1974) (Italy).

## Discussion

### Dryinidae and Encyrtidae collected by field survey in 2014

Through our survey in 2014, two dryinids, *Gonatopus nigricans* (Perkins) and *Haplogonatopus oratorius* (Westwood), emerged from *Laodelphax striatellus* (Fallén) and dryinid cocoons collected from the field. All hyperparasitoids were identified as *Helegonatopus dimorphus*, but a cocoon collected at Hida was parasitized by *Cheiloneurus exitiosus*. We found two other examples of *C. exitiosus* emergence from *Ha. apicalis* parasitizing *Sogatella furcifera* in Saga Prefecture. This species seems to be rare in Japan, or they simply might not prefer *L. striatellus*. On the other hand, *He. dimorphus* was widely found from central Honshu (Saitama and Kanagawa) to Kyushu (Fukuoka, Kumamoto). The rearing of parasitized planthoppers revealed that two dryinid larvae were parasitized by Encyrtidae in Hida (Table 2). The proportion of parasitized field cocoons differed greatly and depended on the site of collection (Table 3). The highest score of 87.9% was recorded in Suya. Since the proportion of *G. nigricans* in Kumamoto was very low (2.1%, compared to 50.0% in Kagoshima), dryinids attacked by encyrtids were regarded as *Ha. oratorius*. No hyperparasitoid was found in Kagoshima.

### Clutch size and sex ratio of *Helegonatopus dimorphus*

Olmi and Xu (2015) reported that 10–12 individuals of *He. dimorphus* emerged from a cocoon of *G. rosellae* (Currado & Olmi, 1974), while 7 individuals were recorded as having emerged from *G. solidus* (Haupt, 1938) in Italy. According to our field survey, 3–8 (mean 4.95±1.40SD) individuals emerged from *Ha. oratorius*. The average male proportion is 0.19 (Table 4), and represents 0.95 per clutch. The smaller number relative to the other two dryinid species may be due to the smaller body size (2.0–3.1 mm for the females of *Ha. oratorius*, compared to 3.0–3.4 mm in *G. solidus* and 3.5–3.8 mm in *G. rosellae*). This number is similar to that of *He. pseudophanes* (Perkins, 1906) in Argentina where they attack *Gonatopus bonaerensis* Virla, 1997 (2.4–3.2 mm) and *G. desantisi* Olmi & Virla, 1992 (2.9–3.2 mm), and exhibit a clutch size of 6 and male proportion of 0.32 (De Santis and Virla 1991).

### Host stage

As for *C. exitiosus*, they attack the "later instar" larva of *G. nudus* (Perkins, 1912) (as *Pseudogonatopus nudus*) (Manickavasagam et al. 2009). According to the ovservations of De Santis and Virla (1991), both genera may have the ability to begin attacking from the larval-sac stage on the primary host to the pupa in the cocoon. In the present study, we confirmed that *He. dimorphus* can attack the larval sac, but the proportion of this parasitism is much smaller than that of the cocoon. The higher proportion of parasitism can be at least partially explained by the difference in developmental period. The developmental period of the larval sac of *Ha. oratorius* (as *Ha. atratus*, 4.5 days at 25°C) is shorter than the period from removal from the host planthopper to adult emergence (11.2 days) (Kitamura 1983). Our results indicate that *L. striatellus* possibly transports its primary and secondary parasitoid together, but the amount of secondary parasitoid should be very limited not only because of their scarcity. Host stage preference should be investigated to understand the effect on host-parasitoid dispersal dynamics.

## Supplementary Material

XML Treatment for Cheiloneurus exitiosus

XML Treatment for Helegoantopus dimorphus

## Figures and Tables

**Figure 1a.**
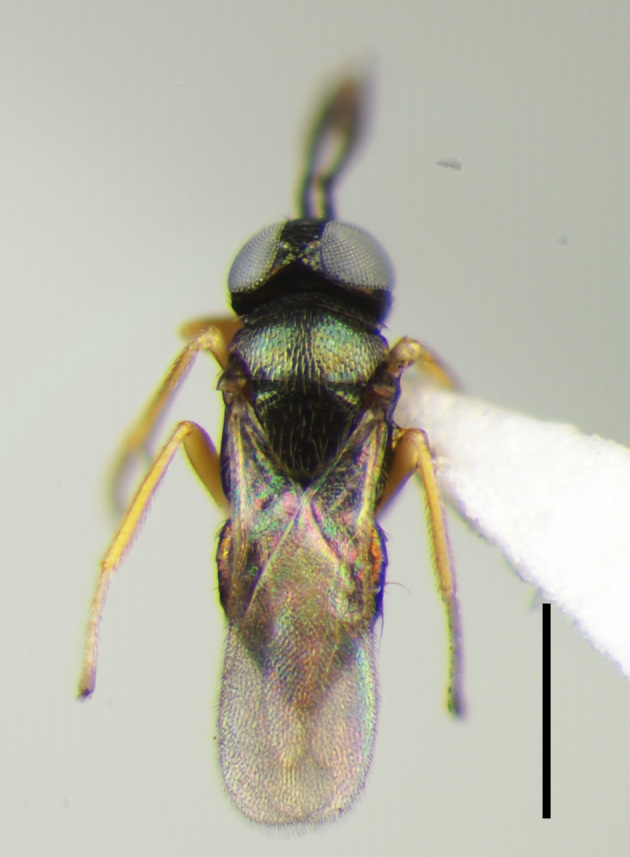
Female.

**Figure 1b.**
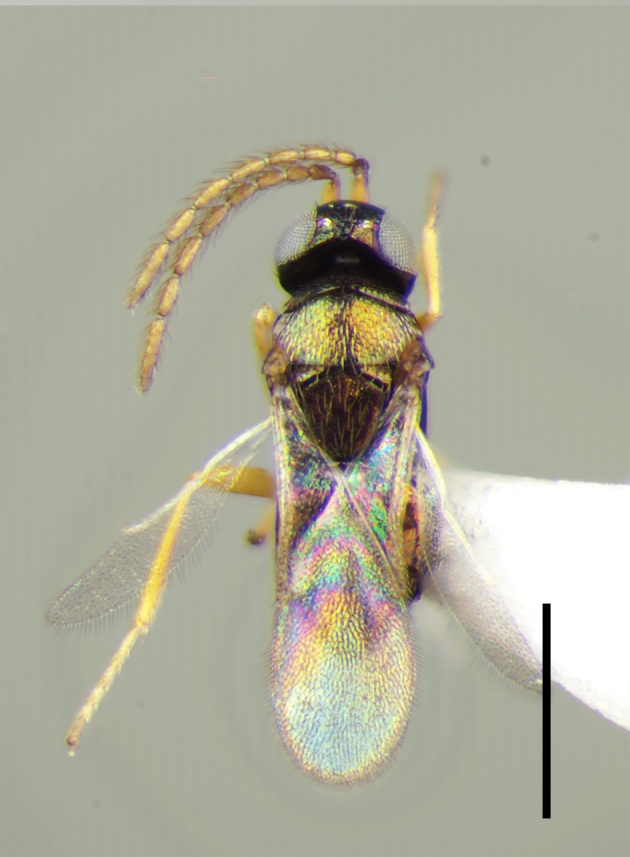
Male.

**Figure 2a.**
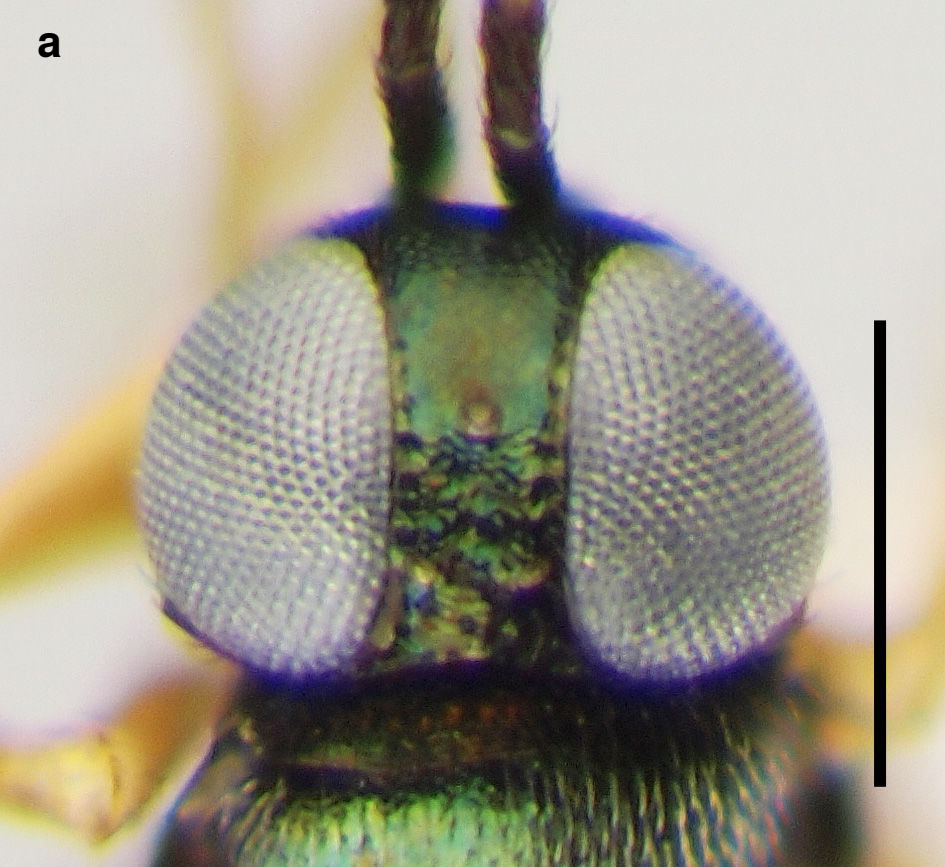
Female.

**Figure 2b.**
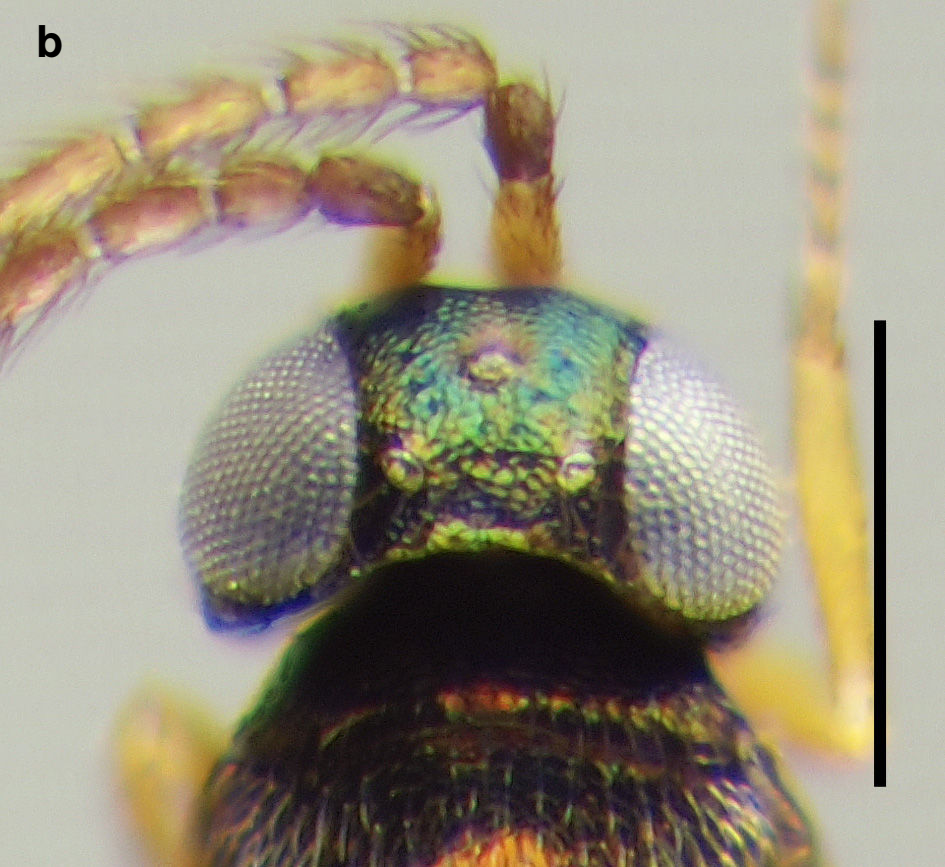
Male.

**Figure 3a.**
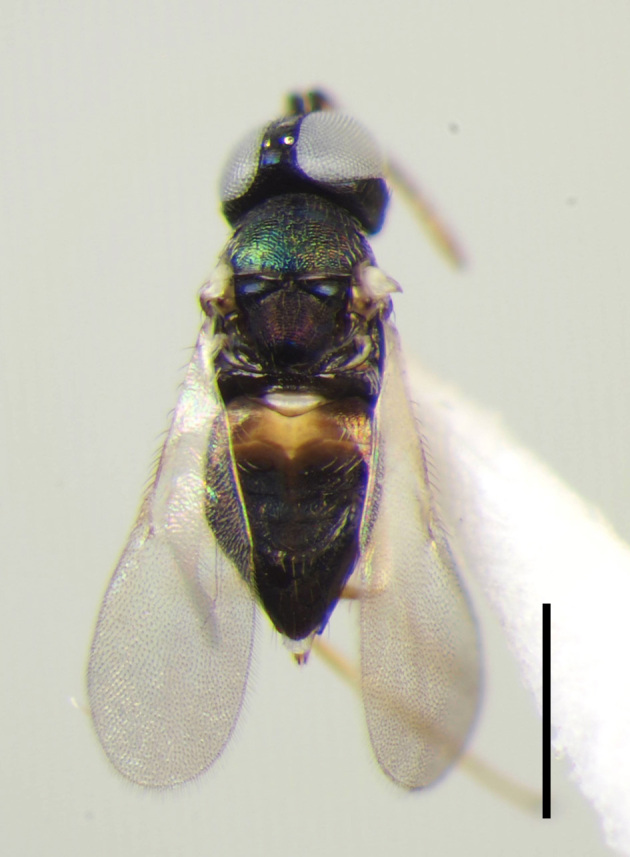
Female.

**Figure 3b.**
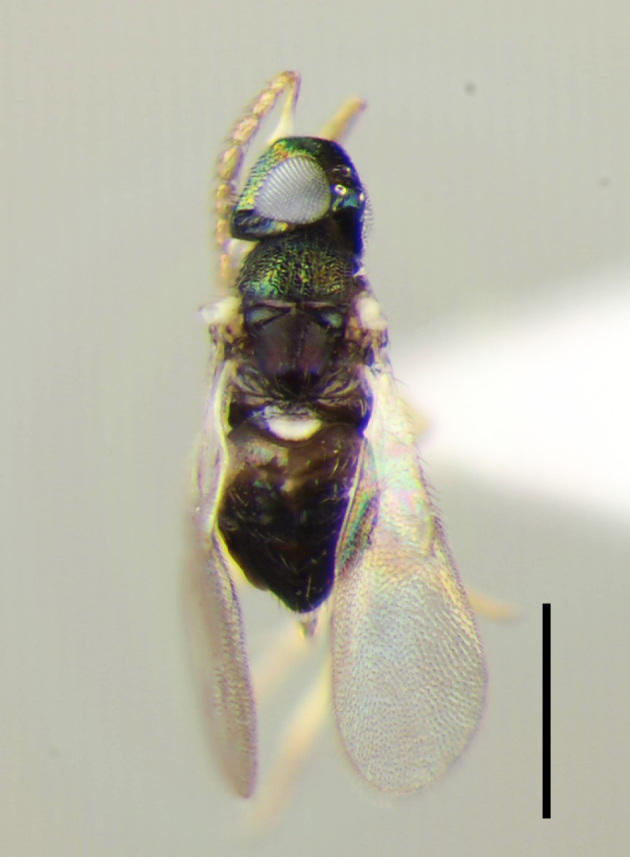
Male.

**Figure 4a.**
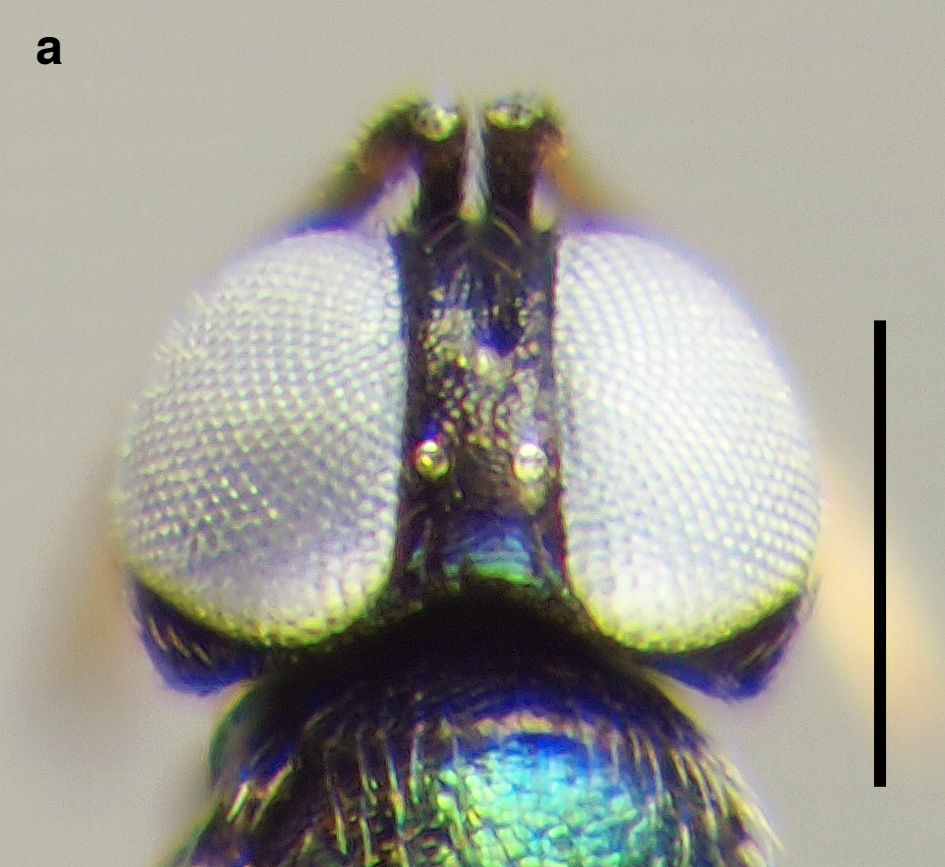
Female.

**Figure 4b.**
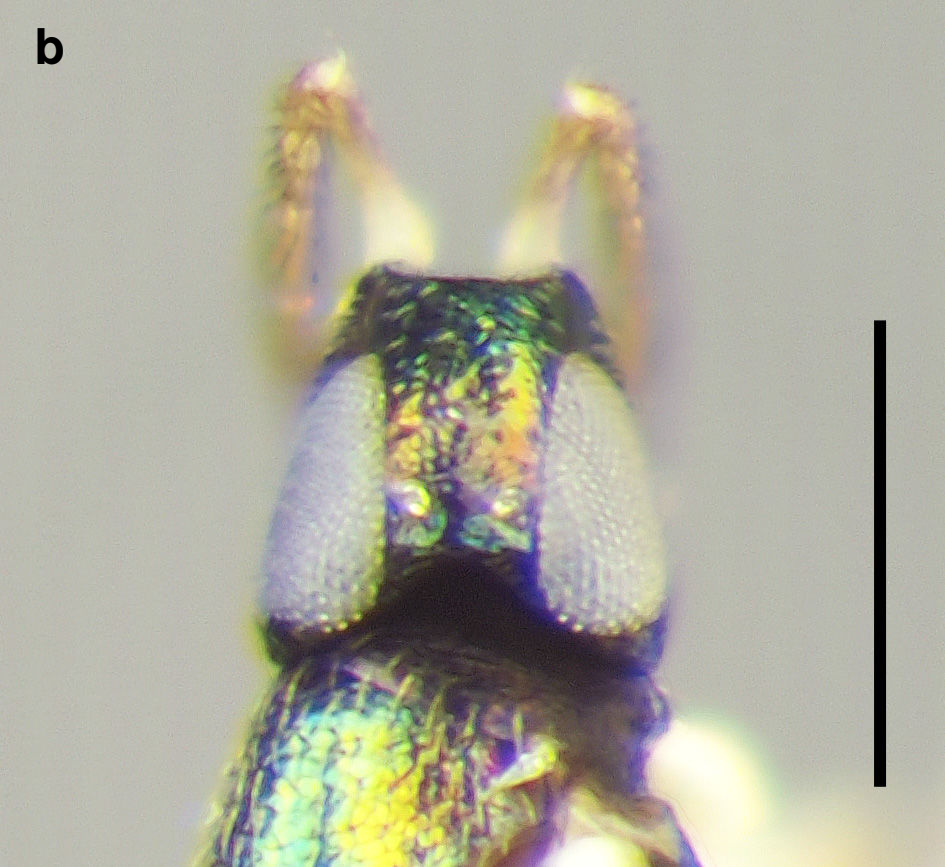
Male.

**Table 1.** Collection sites of field survey in 2014.

Prefecture	Site name	Locality	Latitude	Longitude	Date
Kumamoto	Shisui	Shisui-machi, Kikuchi-Shi	32.927N	130.766E	21. IX. 2014
Kumamoto	Suya	NARO Kyushu Okinawa Agricultural Research Center, Suya, Koshi-Shi	32.876N	130.738E	17. IX. 2014
Kumamoto	Hida	Hida, Kita-ku, Kumamoto-Shi	32.849N	130.720E	18, 20. IX. 2014
Kagoshima	Kimpo	Kagoshima Prefectural Institute for Agricultural Development, Kimpo-cho, Minamisatsuma-Shi	31.482N	130.343E	26-28. IX. 2014

**Table 2.** Parasitized *Laodelphax striatellus* and emerged parasitoids collected by field survey in 2014.

	*Laodelphax striatellus*	*Haplogonatopus oratorius*	*Gonatopus nigricans*	*Helegonatopus dimorphus*	*Cheiloneulus exitiosus*
Suya	3	3	0	0	0
Shisui	30	30	0	0	0
Hida	73	70	1	2	0
Kimpo	54	28	26	0	0

**Table 3.** Field cocoons and emerged parasitoids collected by field survey in 2014.

	Cocoon	*Haplogonatopus oratorius*	*Gonatopus nigricans*	*Helegonatopus dimorphus*	*Cheiloneurus exitiosus*
Suya	33	4	0	29	0
Shisui	26	24	0	2	0
Hida	17	9	2	5	1
Kimpo	2	0	2	0	0

**Table 4.** Two encyrtid species collected by field survey in 2014.

	Clutch number	Emerged number	Sex ratio (male proportion)
*Helegonatopus dimorphus*	38	188	0.19
*Cheiloneulus exitiosus*	1	5	0.20
